# Serial Measurements of Splanchnic Vein Diameters in Rats Using High-Frequency Ultrasound

**DOI:** 10.3389/fphar.2016.00116

**Published:** 2016-05-03

**Authors:** Bridget M. Seitz, Teresa Krieger-Burke, Gregory D. Fink, Stephanie W. Watts

**Affiliations:** ^1^Department of Pharmacology and Toxicology, Michigan State University, East LansingMI, USA; ^2^In Vivo Facility, Michigan State University, East LansingMI, USA

**Keywords:** ultrasound, serial imaging, splanchnic veins, venous capacitance, venous diameter_5_, cardiovascular diseases

## Abstract

The purpose of this study was to investigate serial ultrasound imaging in rats as a fully non-invasive method to (1) quantify the diameters of splanchnic veins in real time as an indirect surrogate for the capacitance function of those veins, and (2) assess the effects of drugs on venous dimensions. A 21 MHz probe was used on anesthetized male Sprague–Dawley rats to collect images containing the portal vein (PV), superior mesenteric vein (SMV), abdominal inferior vena cava (IVC), and splenic vein (SpV; used as a landmark in timed studies) and the abdominal aorta (AA). Stable landmarks were established that allowed reproducible quantification of cross-sectional diameters within an animal. The average diameters of vessels measured every 5 min over 45 min remained within 0.75 ± 0.15% (PV), 0.2 ± 0.09% (SMV), 0.5 ± 0.12% (IVC), and 0.38 ± 0.06% (AA) of baseline (PV: 2.0 ± 0.12 mm; SMV: 1.7 ± 0.04 mm; IVC: 3.2 ± 0.1 mm; AA: 2.3 ± 0.14 mm). The maximal effects of the vasodilator sodium nitroprusside (SNP; 2 mg/kg, i.v. bolus) on venous diameters were determined 5 min post SNP bolus; the diameters of all noted veins were significantly increased by SNP, while mean arterial pressure (MAP) decreased 29 ± 4 mmHg. By contrast, administration of the venoconstrictor sarafotoxin (S6c; 5 ng/kg, i.v. bolus) significantly decreased PV and SpV, but not IVC, SMV, or AA, diameters 5 min post S6c bolus; MAP increased by 6 ± 2 mmHg. In order to determine if resting splanchnic vein diameters were stable over much longer periods of time, vessel diameters were measured every 2 weeks for 8 weeks. Measurements were found to be highly reproducible within animals over this time period. Finally, to evaluate the utility of vein imaging in a chronic condition, images were acquired from 4-week deoxycorticosterone acetate salt (DOCA-salt) hypertensive and normotensive (SHAM) control rats. All vessel diameters increased from baseline while MAP increased (67 ± 4 mmHg) in DOCA-salt rats compared to SHAM at 4 weeks after pellet implantation. Vessel diameters remained unchanged in SHAM animals. Together, these results support serial ultrasound imaging as a non-invasive, reliable technique able to measure acute and chronic changes in the diameter of splanchnic veins in intact rats.

## Introduction

Dysregulation of venous capacitance (venous volume at a given transmural pressure) is proposed to contribute to the etiology of heart failure, arterial hypertension, hepatic cirrhosis, pre-eclampsia, postural hypotension and shock (Safar and London, 1987; Reilly et al., 2001; Stewart et al., 2004; Aardenburg et al., 2005; Li et al., 2008; Burchell et al., 2013). Splanchnic veins, located within the abdominal region, represent the largest blood volume reservoir within the human body (Gelman, 2009), and also exhibit the largest degree of active capacitance response of all venous beds in the body (Tyberg, 2002). Therefore, they play an important role in the regulation of the circulation by affecting cardiac preload, and thus, cardiac output and blood pressure. Early methods to study splanchnic venous capacitance and its regulation were highly invasive and could only be readily applied in experimental animals (Brooksby and Donald, 1971). Later approaches that were applicable to humans included examining the distribution of blood-sequestered radionuclides (Schmitt et al., 2002), impedance plethysmography (Leunissen et al., 1993), and direct imaging of the large veins (Stewart and Montgomery, 2004). For example, ultrasonographic assessment of the dimensions of large veins [particularly the inferior vena cava (IVC)] has been used to estimate venous capacitance as a measure of intravascular volume status in patients with septic shock (Schefold et al., 2010) and in patients on hemodialysis (Brennan et al., 2006).

Recent advancements in ultrasound technology include the development of high-frequency transducers (up to 70 MHz for rodent imaging). The associated enhanced signal processing at rapid frame rates (132 frames/second), with superior resolution^1^, enables quality ultrasound imaging in small animals (Deshpande et al., 2010; Stuart et al., 2011). The specific probe used in this study, the 21 MHz MS250 probe, has a resolution of 75 μM axial by 165 μm lateral^2^. Importantly, rapid real-time imaging allows longitudinal measurements of the structure and function of small structures without significant impact on the animal or its physiology.

While the current literature highlights many uses of high-frequency ultrasound in rodent cardiovascular models (Coatney, 2001; Slama et al., 2003; Chen et al., 2012), imaging of the abdominal veins has not been widely described in animal research. While many rat splanchnic veins can be readily visualized using ultrasound (Knipp et al., 2003), there is no report of a technique that observes several major splanchnic vessels (both veins and arteries) at once with repeated measures of the same location along a specific splanchnic vessel. The ability to re-locate and measure a precise section along a vessel is critical to allow the continuous evaluation of diameter changes of that vessel during pharmacological interventions, or in chronic pathological states. We have therefore developed a technique for serial ultrasound imaging and measurement of the splanchnic vessel diameters in the anesthetized rat with the goal of providing a reproducible, longitudinal, and non-invasive index of the capacitance function of these vessels. Validation of this technique included determining if imaging could reliably detect acute changes (seconds to minutes) in splanchnic vein diameters caused by venoactive drugs. Another focus was testing whether imaging could detect stable changes in the diameter of splanchnic veins in chronic conditions such as hypertension. Finally, this work was essential in developing a tool that would allow interrogation of the role of the venous circulation in drug-induced blood pressure changes. Specifically, our laboratories are dedicated to understanding how serotonin (5-HT, 5-hydroxytryptamine) lowers blood pressure chronically (Diaz et al., 2008). *In vitro* work on isolated splanchnic veins strongly supports the ability of 5-HT to cause direct venodilation but not arterial dilation (Watts et al., 2015). As such, a validated mechanism by which to investigate *in vivo* venous diameter would allow us to connect *in vitro* and *in vivo* studies, a powerful approach when investigating cause and effect. This work suggests the feasibility of using serial ultrasound imaging to investigate venous function in rodents.

## Materials and Methods

### Animals

MSU Institutional Animal Care and Use Committee approved all protocols used in this study. Male Sprague–Dawley rats at 6-weeks of age (300–350 g; Charles River Laboratories,) were used in all experiments. Rats were housed in a temperature–controlled room (22°C) with 12-h light/dark cycles and given standard chow and distilled water *ad libitum*.

### Ultrasound Imaging

**FIGURE 1:**
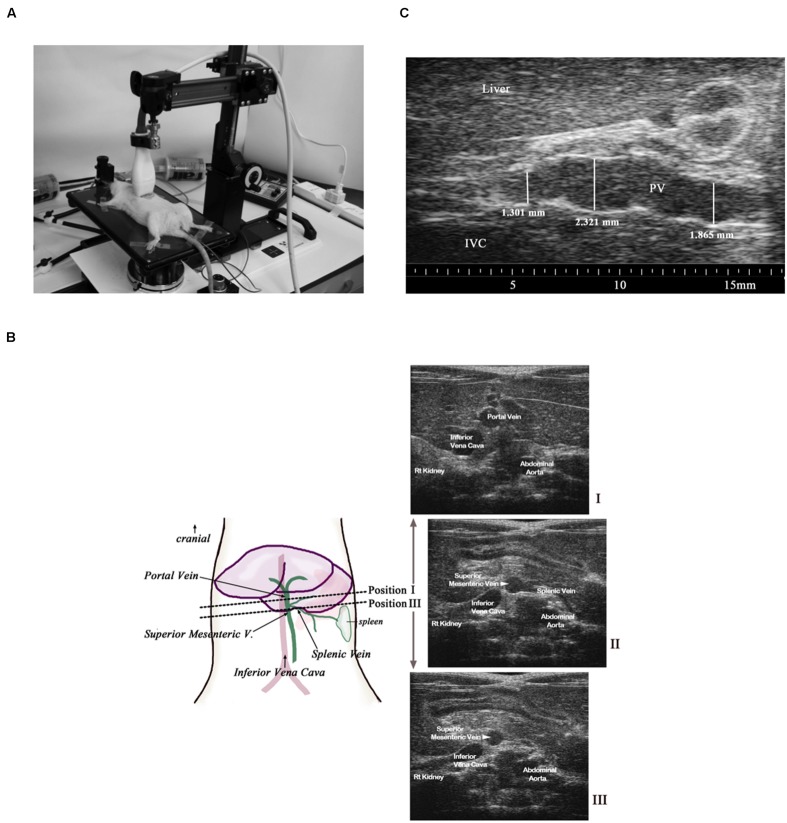
**(A)** A mechanical rail was used to hold a 21 MHz linear probe perpendicular to the abdomen while collecting transverse images. Body temperature was maintained at 37°C. All measurements were made using images collected during expiration and during cardiac systole. **(B)** Transverse transducer placement (dotted line) and corresponding images obtained (Images I, II, and III). The transducer placement to obtain the superior mesenteric vein (SMV)/ SpV view is ∼2 mm caudal to the placement used to obtain the portal vein (PV) view. **(C)** A longitudinal image of the PV within a naïve rat shows the variability of the diameter of this vessel along its length even when controlled for respiration and cardiac cycle.

All animals were anesthetized using isoflurane (1.5–2.5% in oxygen, titrated to maintain stable heart rate (HR), respiration rate, and body temperature). The upper abdomen was shaved and depilatory cream (Nair^TM^) applied below the xiphoid process. Animals were positioned supine on a heated platform [Vevo 2100 Imaging Station (integrated rail system); Visualsonics, Toronto, Canada], as shown in **Figure 1A.** Coupling gel was applied to all four paws and the paws were taped to conductive pads on the platform to allow continuous collection of HR, respiratory rate, body temperature, and electrocardiograms (ECGs) during ultrasound image collection. Body temperature was monitored continuously, via a rectal probe, and maintained at 37°C throughout the experiment using the heated table and when necessary, a supplemental heat lamp. Warmed ultrasound gel was applied to the abdominal skin, just below the xiphoid process to couple the transducer (21 MHz probe; Visualsonics MS250) for imaging. The transducer head was locked in the adjustable arm of the Vevo mechanical rail-system to allow accurate hands-free transducer positioning during image collection. The height of the transducer was set to apply minimal pressure to the abdomen while still allowing adequate views of the abdominal vessels of interest. Once the transducer was positioned, the x–y knobs of the platform were used to move the transducer small increments in the cranial and caudal directions to display the major abdominal vessels significant to this study: abdominal aorta (AA), IVC, and portal vein (PV) in the same view. Transverse B-mode images were collected at 25 frames per second in two locations, as seen in **Figure 1B.** In Image I, we focused on a transverse view at the level of the PV exiting the liver, which provides simultaneous cross-sectional images of the AA, IVC, and PV; these structures are labeled as such. Image II displays the splenic vein (SpV), the established landmark for locating the noted veins. Finally, image III provides a transverse view ∼2 mm caudal to Image I, at a level just below the branching of the SpV (shown in image II), and shows the structures of the superior mesenteric vein (SMV), SpV, and IVC. **Figure 1C** shows a longitudinal image of the PV (as it exits the liver) during systole and expiration. All ultrasound images were saved as cine loops for subsequent measurement and analyses of vessel diameters. This technique was used to collect baseline and post dose images during drug interventions. Ultrasound recordings from individual rats during chronic studies were collected at the same time each day and feeding schedule remained consistent throughout the studies. We assumed venous pressure remained stable during each of these interventions and cardiac function did not change, except in DOCA animals. The AA was used as a control abdominal blood vessel for which minimal dynamic changes in measureable diameter were expected.

### Ultrasound Image Analysis

All vessel diameter measurements were made by the same ultrasonographer with considerable experience in the use of the Vevo Imaging system. Cine loops of the images were viewed on a desktop station using Vevo LAB 1.7.1 software (Visualsonic, Toronto, Canada). All vessel diameters were measured using image frames from periods of cardiac systole and during expiration. Both vertical and horizontal diameters (vessel height and vessel width) of vessel cross-sections were measured and then averaged to obtain final values from each time point.

### Time Course Studies

We performed two time course studies: (1) images were collected for three animals every 5 min over the course of 45 min (acute); (2) images were collected for five animals every 2 weeks over the course of 8 weeks (chronic).

### Blood Pressure Monitoring

Rats used in the sodium nitroprusside (SNP) and sarafotoxin experiments were anesthetized with isoflurane (2% in oxygen) and a femoral arterial catheter was implanted for blood pressure monitoring. The catheter was placed through a 1–1.5 cm incision in the left inguinal area into the left femoral artery immediately prior to ultrasound imaging. The tip of the catheter was advanced to the AA and the catheter was attached to a transducer connected to a data acquisition system (Powerlab, ADinstruments) to record mean arterial pressure (MAP) and HR during imaging. Rats remained anesthetized throughout catheter placement and subsequent imaging.

### Vasodilation Study- Sodium Nitroprusside (SNP)

After 3 min of baseline recordings of abdominal vessel images and blood pressure, each animal received a 2 mg/kg bolus (0.3 mL, tail vein) of SNP (5054, Sigma Chemical CO., St Louis, MO, USA). Ultrasound images were collected every minute over 30 min with 30 min being the time at which MAP returned to near baseline values. Data were collected at 5 min post-bolus, the point at which the MAP had reach nadir while vessel diameters initial achieved near maximum dilation during the 30 min of the experiment. These maximum changes were reported graphically. MAP was measured continuously via a femoral arterial catheter connected to data acquisition software on an ADInstruments Powerlab (Chart 7.0, Colorado Springs, CO, USA). The SpV was not used as an endpoint in this particular study, but solely as a landmark. Seven animals were used in this study.

### Vasoconstriction Study- Sarafotoxin (S6c)

After 3 min of baseline recordings of abdominal vessel images and blood pressure, each animal received a 5 ng/kg bolus (0.3 mL, tail vein) of S6c (88-9-35, American Peptide, Sunnyvale, CA, USA). Ultrasound images were collected every minute over 15 min at which time all imaged vessels and MAP had returned back to baseline values. Data was graphed at 5 min’ post-bolus, the point of maximum vessel diameter change. MAP was measured continuously via a femoral arterial catheter connected to data acquisition software on an ADInstruments Powerlab (Chart 7.0, Colorado Springs, CO, USA). The SpV was not used as an endpoint in this particular study, but solely as a landmark. Five animals were used in this study.

### Deoxycorticosterone Acetate-Salt Study (DOCA-Salt)

All rats used during the DOCA-salt study underwent a left uninephrectomy. A DOCA pellet was implanted subcutaneously in half of the rats to deliver the drug at a dose of 200 mg/kg. The other rats underwent a SHAM pellet surgery (SHAM group). All rats were switched from distilled drinking water, to water containing 1% NaCl plus 0.2% KCl for 4 weeks. All rats were imaged at baseline (prior to any intervention), and again 4 weeks after surgery/pellet implantation. Systolic arterial pressure was measured 4 weeks after surgery in conscious rats via the tail cuff method (Malkoff, 2005; Kent Scientific Corp, CODA 6); this was the only time blood pressure was measured. There were four animals in each group: DOCA-salt and SHAM.

### Data Analysis

Values were either expressed as mean ± SEM of the number of animals in the experiment, or a percent change from baseline. The baseline was determined by 3 min of image collection, prior to injection or during the 1st day of imaging prior to treatment. Statistical analyses were performed using paired two-tailed *t*-tests when comparing vessel images to baseline values. A repeated measures ANOVA were performed when comparing vessel diameter values at different time points in timed studies. (GraphPad Prism 6). In all cases, a *p*-value of <0.05 was considered significant.

## Results

### Ultrasound Transducer Placement

The splanchnic vein diameters varied considerably along the length of those vessels within the same rat as shown in **Figure 1C** and it was therefore imperative that a landmark be identified to allow repeated placement of the transducer at the exact location along a particular vein. Preliminary work suggested the SpV could be used as a landmark to locate and differentiate the PV and the SMV as shown in **Figure 1B**, image II. Because it was important to visualize the branching of the SpV off the parent vessels and to include multiple vessels of interest within the same view, a transverse positioning of the ultrasound transducer as seen in **Figure 1A** was determined to provide the optimal views for measurements of the PV, SMV, SpV, IVC, and AA diameters. In addition, the use of high-frequency ultrasound allowed the collection of images throughout the cardiac and respiratory cycles despite the rapid heart and respiratory rates of rats. Data could be generated from vessels during systole and exhalation specifically.

### Acute Time Course Study

**FIGURE 2:**
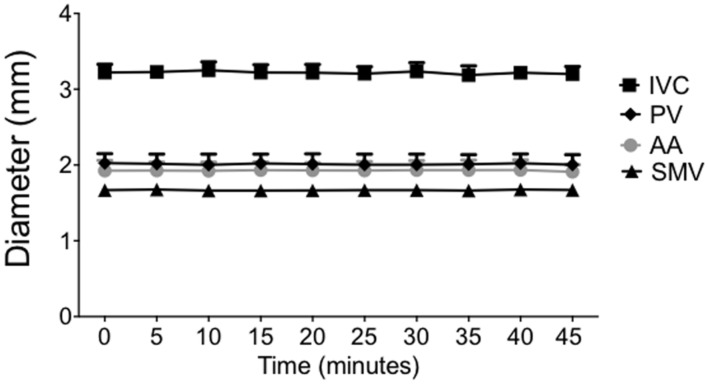
**Under isoflurane anesthesia, animals were imaged every 5 min over 45 min to determine the stability and consistency of each vessel diameter.** Points on the graph represent vessel diameters (means ± SEM of three animals).

Vessel diameters measured every 5 min in anesthetized rats were unchanged with no statistical difference from baseline diameters through at least 45 min as seen in **Figure 2.** The diameters remained within 0.75 ± 0.15% (PV), 0.2 ± 0.09% (SMV), 0.5 ± 0.12% (IVC), and 0.38 ± 0.06% (AA) of baseline measurement for each noted vessel. The SpV was not imaged in this particular study.

### Acute Pharmacologic Intervention Studies

**FIGURE 3:**
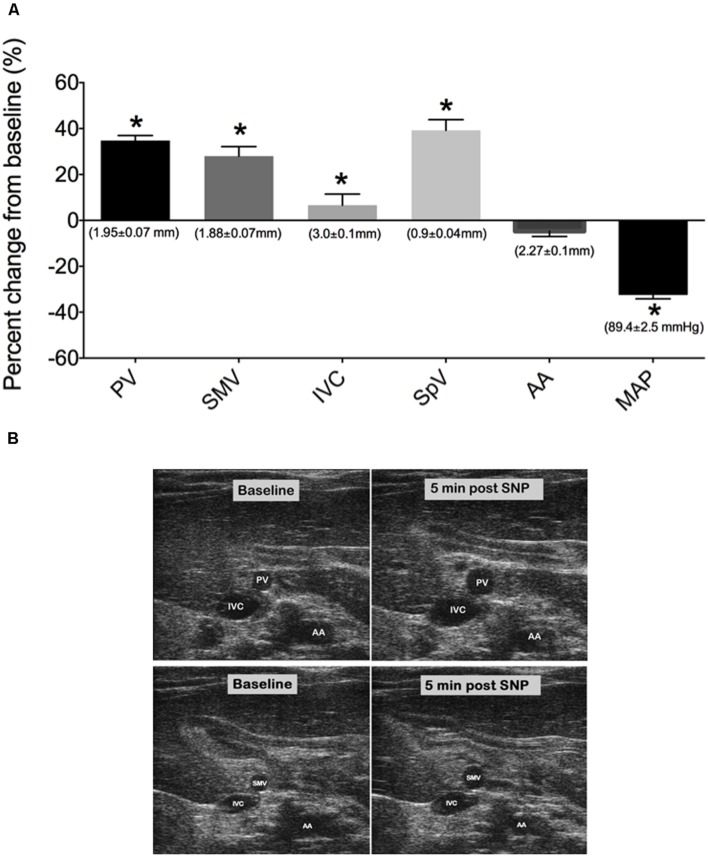
**(A)** Percent change from baseline (baseline measured 3 min prior to drug treatment) of vessel diameters and MAP at 5 min post SNP i.v bolus. Bars represent means ± SEM of seven animals. Asterisks depicts statistically different from baseline, *P* < 0.05. **(B)** Images of PV, IVC, SMV, and AA at baseline and 5 min post SNP. Baseline diameters of vessels are shown in the foundation of each bar.

**FIGURE 4:**
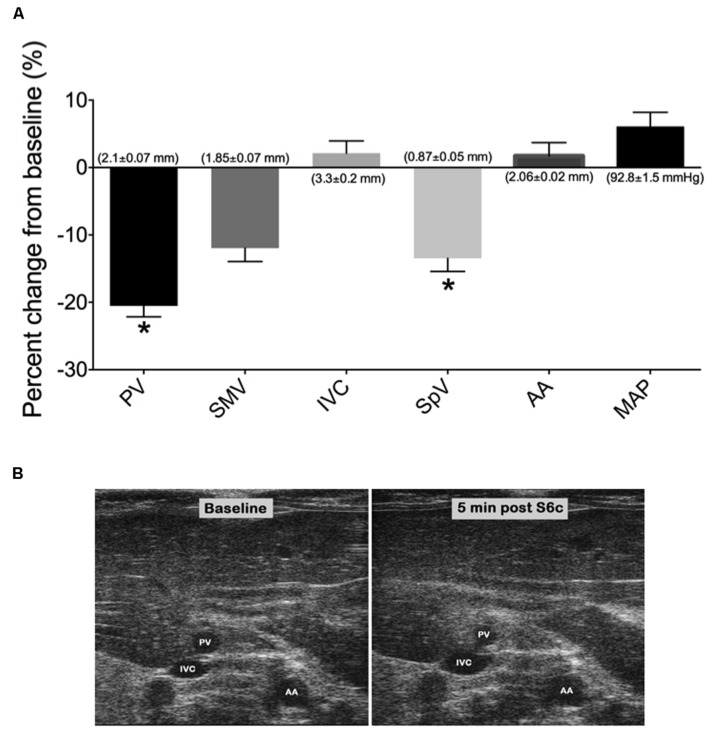
**(A)** Graph represents the percent change from baseline (measured 3 min prior to drug treatment) of vessel diameters and MAP at 5 min post S6c i.v bolus. Bars represent means ± SEM of five animals. Asterisks depicts statistically difference from baseline, *P* < 0.05. **(B)** Images of PV, IVC, and AA at baseline and 5 min post S6c. Baseline diameters of vessels are shown in the foundation of each bar.

The venodilator SNP and the venoconstrictor S6c were used in separate experiments to investigate the ability of our imaging method to detect acute drug-induced changes in venous diameter. A single iv bolus of SNP at 2 mg/kg caused significant peak increases in PV, SMV, SpV, and IVC diameters by 5 min post drug administration as seen in **Figures 3A,B**; (PV: 34.70 ± 2.2%, SMV: 28 ± 4.1%, SpV: 39.3 ± 4.7%, and IVC: 6.6 ± 4.8% change from baseline). Actual diameters of vessels at baseline are reported in the foundation of each bar. In contrast, there was little change in the diameter of the AA to SNP despite MAP being significantly reduced by 29 ± 4 mmHg. In separate rats, a single 5 ng/kg iv bolus of S6c caused a peak decrease in the diameters of the SMV (11.9 ± 2.0%), PV (20.5 ± 1.6%), and SpV (13.5 ± 2%) 5 min after injection, while MAP increased 6 ± 2 mmHg as shown in **Figures 4A,B.** The percent change from baseline in vein diameter for both PV and SMV in the presence of S6c were statistically significant (*p*-value of <0.05) with SMV (*p*-value 0.067) and MAP (*p*-value 0.105) close to significance. The SMV image was not included in **Figure 4B** since SMV did not show a statistical significance in the presence of sarafotoxin. There was little change in the diameters of the IVC or AA. Baseline diameters are reported in the foundation of each bar.

In addition, the arterial catheter which was placed in the femoral artery to measure MAP, during each acute pharmacologic intervention study, had no significant affect on the diameter of the PV, SMV, or AA when compared to similar imaged vessels without an arterial catheter (data not shown). There was a modest reduction in the IVC diameter of animals with (3.13 ± 0.11 mm) and without an arterial catheter (3.34 ± 0.05 mm; *p* < 0.05). These differences could possibly be attributed to the larger variability in the diameter size of the IVC, or be a response to occlusion of the femoral artery during catheter insertion. It is important to note, measurements were always compared to baseline, as our focus was on change from baseline rather than actual diameter changes.

### Chronic Time Course Study

**FIGURE 5:**
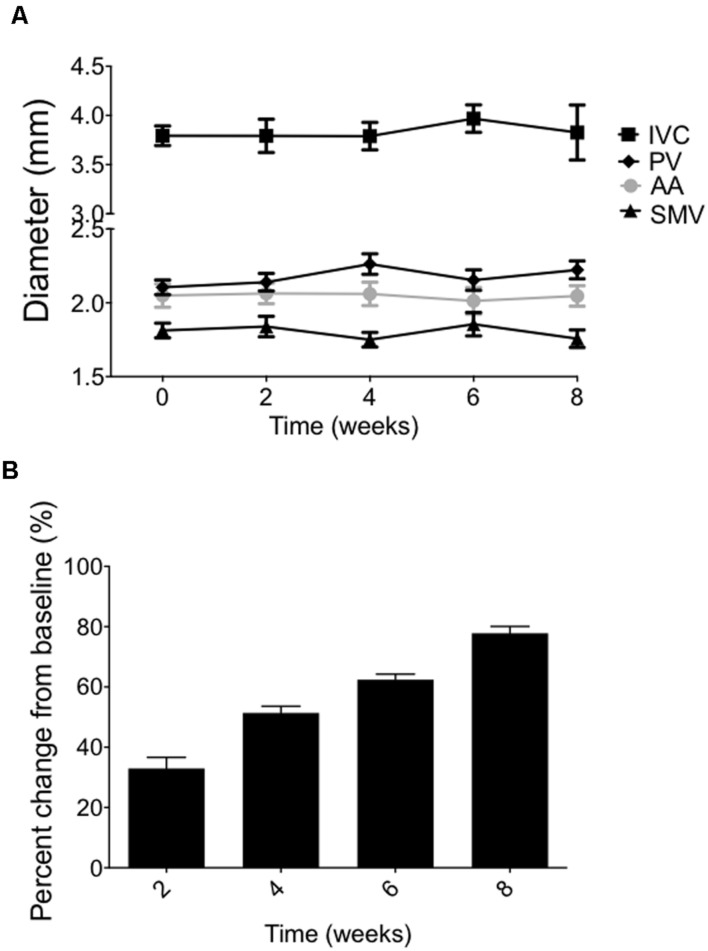
**(A)** Vessel diameters imaged every 2 weeks through 8 weeks (means ± SEM of five animals). **(B)** Body weight changes of the same animals over the 8-week time course. Bars represent means ± SEM of five animals.

In a longitudinal imaging study, we evaluated the stability of the splanchnic veins and AA diameters measurements within an animal when acquired every 2 weeks, over 8 weeks. Vascular diameters measured using our imaging technique were extremely stable over 8 weeks, showing the average of the percent changes from baseline at 8 weeks to be within 2.0 ± 0.5% (PV), 0.95 ± 0.2% (SMV), 11.7 ± 8% (IVC), and 0.9 ± 0.6% (AA), as shown in **Figure 5A.** The SpV was not imaged in this particular study. There was no statistical difference between the time points of the imaged vessels. These findings support the ability to consistently relocate a specific cross-section of abdominal vessel of interest over multiple imaging sessions spanning many weeks within an individual animal. Interestingly, vessel diameters remained stable despite a steady body weight gain (200 g) over the course of the experiment (**Figure 5B**). All animals used in these studies were mature rats (10–12 weeks of age at the start of each experiment) with stable body lengths, however.

### Deoxycorticosterone Acetate-Salt Study (DOCA-Salt)

**FIGURE 6:**
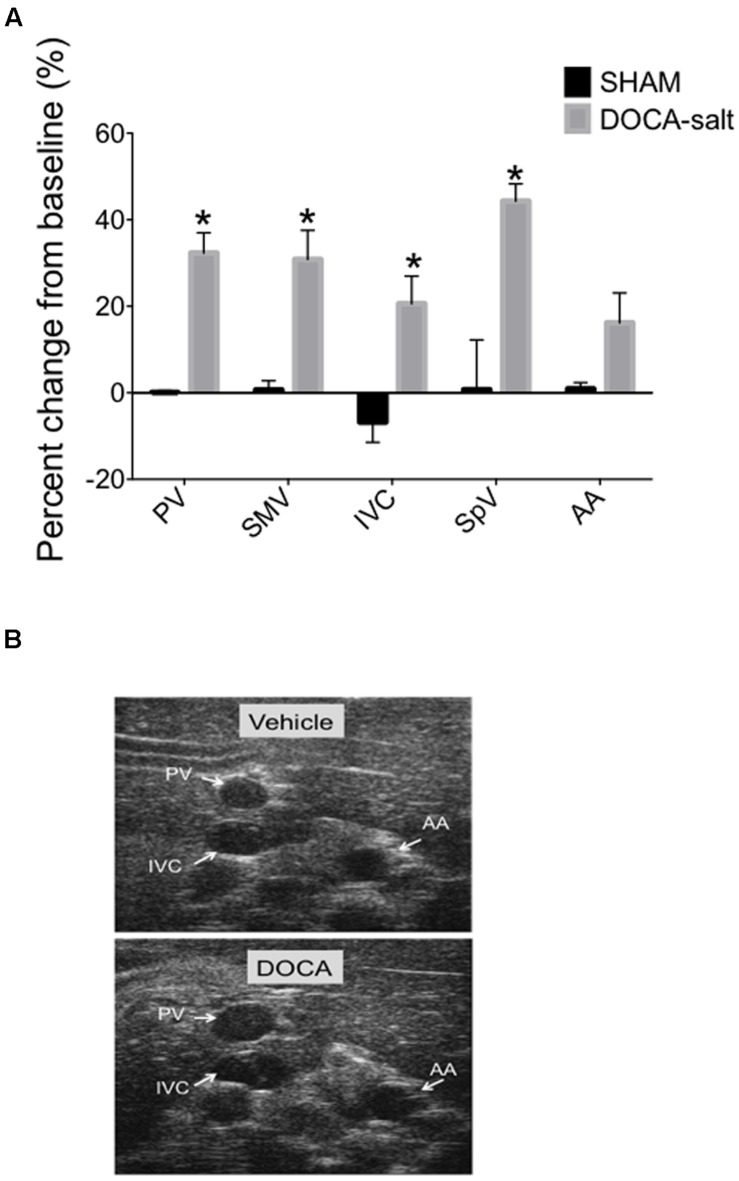
**(A)** Percent change from baseline (baseline measured prior to DOCA-salt treatment) of vessel diameters compared to 4-weeks post DOCA-salt treatment. Bars represent means ± SEM of four animals per treatment group. Asterisks depict statistically difference from baseline, *P* < 0.05. **(B)** Images of PV, IVC, and AA at baseline and 4-weeks post DOCA-salt treatment.

We applied our imaging method to an experimental model of hypertension, DOCA-salt hypertension, in which we have previously demonstrated abnormalities of venous capacitance regulation (Fink et al., 2000). The current experiment involved ultrasound imaging of splanchnic vessels at baseline and again after 4-weeks of established DOCA-salt hypertension. While vascular diameters were similar in the two groups of rats at baseline prior to treatment (less than a 4% difference in any vessel diameters between groups prior to treatment), we observed an overall increase in the vessel diameters of DOCA-salt treated animals compared with vehicle controls at 4 weeks of treatment as quantified and imaged in **Figures 6A,B.** Diameters increased significantly from baseline in the DOCA-salt treated group, 32.3 ± 4.6% (PV), 30.8 ± 6.7% (SMV), 20.6 ± 6.4% (IVC), 44.3 ± 4% (SpV), but not in the AA 16.2 ± 6.9% of the DOCA-salt treated group or vehicle group, 0.1 ± 0.4% (PV), 0.7 ± 2.1% (SMV), −6.8 ± 4.7% (IVC), 0.7 ± 11.5% (SpV), and 0.9 ± 1.4% (AA). Tail-cuff blood pressure was 67 ± 4 mmHg higher in DOCA-salt versus SHAM rats.

## Discussion

The goal of the present study was to develop and validate a non-invasive imaging method to make reliable and reproducible serial measurements of splanchnic vein diameters in anesthetized rats as an aid to understanding the role of vascular capacitance in cardiovascular regulation. We investigated several different vein segments (PV, SMV, IVC, and SpV) because multiple veins contribute to splanchnic vascular capacitance function, and each segment is unique in terms of structure and contractile regulation (Schmitt et al., 2002). Our approach to this goal featured the use of: high frequency ultrasound; an adjustable arm and mechanical rail-system to allow for accurate, stable, hands-free transducer positioning during image collection; identification of reliable anatomical landmarks to facilitate reproducible transducer location; and the measurement of vessels during systole and expiration only (as cardiac and respiratory cycles strongly influence the dimensions of the compliant abdominal veins). The PV, SMV, and SpV all showed marked changes when rats were infused with either S6c or SNP, while the IVC remained relatively stable. This may occur because the effectors of S6c and SNP are differently functional in these compared veins (e.g., is the amount of ET_B_ receptors different, is the expression of guanylate cyclase different, respectively?). Alternatively, diameter changes to the PV, SMV, and SpV may lead to compensatory changes in the IVC that mask effects that might be seen in an isolated IVC. The varying responses of specific vessels *in vivo* highlight the importance of simultaneous and collective diameter measurements.

Our initial study showed that use of this approach produced quite stable measurement of vessel diameters over 45 min in anesthetized, temperature-controlled rats. The stability of the vessel diameters over this time period, in the absence of any intervention, allowed us to conduct subsequent experiments to examine the acute effects of venoactive drugs on splanchnic vessel diameters. Administration of the venodilator SNP and the venoconstrictor S6c resulted in anticipated increases and decreases in splanchnic venous diameters, respectively. These two compounds were chosen because of their relative venoselectivity. This difference in reactivity highlights one of the many differences between veins and arteries, including veins possessing lower smooth muscle content and exposure to lower levels of cyclic strain (Sung et al., 2007). In isolated rat veins and arteries, S6c causes a venoconstriction but not an arterial constriction (Tykocki et al., 2009). This was the best tool we had available that would permit relatively selective venoconstriction and, used with SNP as a recognized vasodilator, demonstrates that we could observe and quantify both venous constriction and dilation. It is important to note that we did not measure pressure within the abdominal veins during image acquisition; therefore firm conclusions cannot be drawn about whether the observed diameter changes were primarily passive or active in nature. However, in a previous study (Schwarzacher et al., 1992) in rabbits that reported changes in the diameter of the abdominal vena cava in response to venoactive drugs, only very small alterations in venous pressure were observed. Thus the diameter changes we observed were likely due to active alterations in venous smooth muscle tone.

In order to validate this imaging technique for use in more chronic longitudinal studies, in a further experiment we investigated the stability of the splanchnic vein and AA diameters within an animal when acquired every 2 weeks over an 8 weeks period. The key to this approach was the ability to accurately relocate a specific cross-section of the splanchnic vessel of interest. The consistency of vessel diameter measurements observed in this chronic time course study indicates that these vessels can serve as their own control in experiments examining the potential impact of interventions on long-term changes in vascular diameter. Therefore, we applied our imaging technique to study splanchnic venous diameters in an experimental model of chronic hypertension, i.e., DOCA-salt hypertension. The results showed a significantly increased diameter of all the splanchnic veins in DOCA-salt rats, whereas no increases were observed in SHAM rats. We have previously reported increased venoconstriction in conscious DOCA-salt rats (Fink et al., 2000) so anticipated that we would observe reduced splanchnic venous diameters in these animals. However, venomotor tone in splanchnic veins is primarily controlled by sympathetic activity, and isoflurane anesthesia is known to dramatically reduce sympathetic activity (Bencze et al., 2013). So the increases in venous diameters we observed in DOCA-salt rats under anesthesia are likely due to passive effects (unopposed by sympathetic venoconstriction) of the volume expansion and cardiac hypertrophy that occur in chronic DOCA-salt rats (Kobrin et al., 1990; Titze et al., 2006). This experiment highlights the complexities of evaluating moment-to-moment regulation of venous diameter; and indicates the desirability of using multiple, and minimally invasive, approaches to that evaluation. Nevertheless, the results support the ability of our vein imaging technique to detect chronic, physiologically significant changes in venous diameter in intact (albeit anesthetized) rats.

There are some additional limitations to the current study that deserve mention. First, we only studied a relatively small number of male Sprague–Dawley rats between 6 and 20 weeks of age. Venous diameters measured by ultrasound vary by age and sex in humans (Kutty et al., 2014; Gui et al., 2015) and are likely to do so in rats as well. Likewise, it is quite possible that the approach described here may need to be modified if applied to other strains of rats, to female rats, or to sexually immature or old rats. Second, although we found that weight gain of ∼200 g (∼80% increase) did not significantly affect the diameters of any abdominal vein, studies in humans suggests that venous diameter correlates modestly with body surface area and height (Gui et al., 2015). Third, all animals were imaged only in one spatial plane, i.e., supine. It is possible that altering the posture of the rat would result in different findings for splanchnic vein diameters. Four, since each vessel of interest courses a distinct 3-dimensional plane, it is acknowledged that the combined image may not represent exact perpendicular views of all three vessels at the same time. However, obtaining a single images depicting all three vessels makes possible the evaluation of simultaneous changes to these various vessel sizes that is not possible with constant repositioning of the transducer over each separate vessel. This includes the important ability of being able to directly compare and contrast arterial vs. venous responses. Finally, a relevant consideration for any measurement technique is the time and effort required to master it (Bowra et al., 2015) but since all values here were obtained by a single highly experienced rodent ultrasonographer, we cannot comment on how long it would take for a novice to achieve the kind of stable, reproducible measurements we report.

## Conclusion

Although, ultrasound imaging the abdominal vessels, particularly the PV and IVC, is not novel in experimental research or clinical settings, investigation of diameter change in multiple splanchnic vessels is novel as they relate to venous capacitance. There is no current publication that measures vein diameters in the splanchnic vasculature, a highly capacitive and compliant region important in blood pressure regulation. We anticipate that this technique will be useful in a number of different ways. The goal of the present study was to lay the foundation for repeatable measures, and illustration of changes in one model in which alterations in venous function have been suggested (e.g., the DOCA-salt model). Once we, and hopefully others, accept this type of approach than we can begin to interrogate disease models that would benefit from investigate of the venous circulation. These include other models of experimental and genetic hypertension, hyperlipidemia, obesity and heart failure.

## Author Contributions

SW devised original idea, helped with data analysis, draft revisions, read the final version of the manuscript and worked on this revision. This work was funded by a grant of which she is PI. BS planned and performed experiments, analyzed data and was in charge of pulling this manuscript together (including making figures, etc). She contributed significantly to this revision. TK-B planned and performed experiments, analyzed data, and prepared figures, commented on revisions and final drafts. She contributed significantly to this revision. GF helped with ideas, analysis, experimental planning, working on drafts, and read the final version. He contributed significantly to this revision.

## Conflict of Interest Statement

The authors declare that the research was conducted in the absence of any commercial or financial relationships that could be construed as a potential conflict of interest.

## Footnotes

1 http://www.visualsonics.com/products/vevo-2100

2 www.visualsonics.com/customer/docs/product_info_vevo2100/Vevo_2100-Transducers_v2.8.pdf.
